# Intraoperative Neurophysiological Monitoring in Syringomyelia Surgery: A Multimodal Approach

**DOI:** 10.3390/jcm12165200

**Published:** 2023-08-10

**Authors:** M. Ángeles Sánchez Roldán, Dulce Moncho, Kimia Rahnama, Daniela Santa-Cruz, Elena Lainez, Daniel Baiget, Ivette Chocrón, Darío Gándara, Agustín Bescós, Juan Sahuquillo, María A. Poca

**Affiliations:** 1 Department of Clinical Neurophysiology, Vall d’Hebron University Hospital, Passeig Vall d’Hebron 119-129, 08035 Barcelona, Spain; dulcemaria.moncho@vallhebron.cat (D.M.); kimia.rahnama@vallhebron.cat (K.R.); danielaisabel.santacruz@vallhebron.cat (D.S.-C.); daniel.baiget@vallhebron.cat (D.B.); 2 Neurotraumatology and Neurosurgery Research Unit, Vall d’Hebron Institut de Recerca (VHIR), Vall d’Hebron Barcelona Hospital Campus, Passeig Vall d’Hebron 119-129, 08035 Barcelona, Spain; sahuquillo@neurotrauma.net; 3 Department of Anesthesiology, Vall d’Hebron University Hospital, Passeig Vall d’Hebron 119-129, 08035 Barcelona, Spain; ivette.chocron@vallhebron.cat; 4 Department of Neurosurgery, Vall d’Hebron University Hospital, Passeig Vall d’Hebron 119-129, 08035 Barcelona, Spain; dario.gandara@vallhebron.cat (D.G.); agustin.bescos@vallhebron.cat (A.B.); 5 Department of Surgery, Universitat Autònoma de Barcelona, Bellaterra, 08193 Barcelona, Spain

**Keywords:** Chiari malformation, corticospinal tract mapping, dorsal column mapping, D-wave, intraoperative neurophysiological monitoring, motor evoked potentials, root mapping, somatosensory evoked potentials, spinal cord, syringomyelia

## Abstract

Syringomyelia can be associated with multiple etiologies. The treatment of the underlying causes is first-line therapy; however, a direct approach to the syrinx is accepted as rescue treatment. Any direct intervention on the syrinx requires a myelotomy, posing a significant risk of iatrogenic spinal cord (SC) injury. Intraoperative neurophysiological monitoring (IONM) is crucial to detect and prevent surgically induced damage in neural SC pathways. We retrospectively reviewed the perioperative and intraoperative neurophysiological data and perioperative neurological examinations in ten cases of syringomyelia surgery. All the monitored modalities remained stable throughout the surgery in six cases, correlating with no new postoperative neurological deficits. In two patients, significant transitory attenuation, or loss of motor evoked potentials (MEPs), were observed and recovered after a corrective surgical maneuver, with no new postoperative deficits. In two cases, a significant MEP decrement was noted, which lasted until the end of the surgery and was associated with postoperative weakness. A transitory train of neurotonic electromyography (EMG) discharges was reported in one case. The surgical plan was adjusted, and the patient showed no postoperative deficits. The dorsal nerve roots were stimulated and identified in the seven cases where the myelotomy was performed via the dorsal root entry zone. Dorsal column mapping guided the myelotomy entry zone in four of the cases. In conclusion, multimodal IONM is feasible and reliable and may help prevent iatrogenic SC injury during syringomyelia surgery.

## 1. Introduction

Syringomyelia is the development of a fluid-filled cavity located within the spinal cord (SC) that can increase over time and lead to irreversible neurological damage. Lesions are usually located between C2 and Th9 but can extend up to the brainstem (syringobulbia) or descend to the conus medullaris. The most common etiology of the syringomyelic cavity is a congenital deformity of the craniocervical junction, mainly Chiari malformation (CM). However, it can also be related to prior meningitis, arachnoiditis, spinal trauma, or SC tumors, among others. In addition, idiopathic cases have also been described [1]. The estimated prevalence varies from 1.94 per 100,000 in Japan to 8.9 per 100,000 in Western countries [2].

In syringomyelia cases with a clear etiology that obstructs the normal flow of cerebrospinal fluid (CSF), it has been demonstrated that restoring regular CSF flow by decompressing the SC is more effective and safer than other treatments. For the remaining patients presenting idiopathic syringomyelia or persistent neurological deterioration, apart from the first line of treatment, direct intervention to drain the syrinx via different techniques is accepted as a second-line treatment [3]. There are four main types of direct syringomyelia treatment, including simple syringostomy and three classic types of shunting: syringo-subarachnoid, syringo-peritoneal, or syringo-pleural. However, these strategies are yet to be proven superior in efficacy [4].

Syringostomy, or placing a catheter into the syringomyelic cavity, requires a myelotomy and poses a significant risk of iatrogenic SC injury. Different myelotomy approach techniques for shunt placement have been described, with the dorsal root entry zone (DREZ) and the dorsal median sulcus being the most used surgical entry points. Nevertheless, each method carries specific risks of iatrogenic damage to different SC neural structures.

Intraoperative neurophysiological monitoring (IONM) of the functional integrity of SC pathways is crucial to detect and prevent surgically induced injury. The available methods can be divided into monitoring and mapping techniques. Monitoring modalities, such as motor evoked potentials (MEPs), D-wave, and somatosensory evoked potentials (SEPs), are used to continuously assess the functional integrity of the tracts and are a well-established tool for spine and SC surgery [5,6,7,8]. Mapping techniques allow for the identification of neural structures and, therefore, guide the surgeon in establishing a selective and safer entry point into the SC. In addition to neural root mapping, which is widely established, different mapping methods for SC long tracts—dorsal column (DC) [9,10,11], and corticospinal tract (CST) [12,13,14,15,16]—have been described in the last few years. Dorsal column mapping (DCM) has become a reliable technique for intraoperative midline localization during posterior myelotomy to minimize DC dysfunction syndrome [10,11,17]. All these IONM techniques have been widely described in the literature for various pathologies. However, there are few reports regarding the specific role of IONM in syringomyelia surgery: a surgical procedure that poses a high risk of iatrogenic injury. Here, we evaluate the benefits of multimodal IONM in syringomyelia surgery, including monitoring and mapping techniques, in a cohort of patients with syringomyelia. We highlight the critical role of IONM in selecting a safe myelotomy entry zone, detecting impending damage, and avoiding permanent injuries via adapting the surgical approach according to the IONM data.

## 2. Methods

This retrospective single-center study includes nine patients who underwent surgery to treat syringomyelia of different etiologies using IONM between April 2016 and June 2021. One patient underwent two surgical procedures. The case numbers were assigned chronologically by the date of surgery. The intraoperative neurophysiological data, neurological examination, and pre- and postoperative neurophysiological evaluations were reviewed and analyzed.

### 2.1. Pre- and Postoperative Neurophysiological Evaluations

#### 2.1.1. Electromyography

Electromyography (EMG) was performed using concentric needles in upper and lower limb muscles (deltoid, biceps brachii, triceps brachii, extensor digitorum communis, and first dorsal interosseous, quadriceps, tibialis anterior, and gastrocnemius). Neurogenic changes were identified when muscle unit potentials showed increased amplitude and duration, and acute denervation was diagnosed if spontaneous activity was found.

#### 2.1.2. Somatosensory Evoked Potentials

Somatosensory evoked potentials (SEPs) were measured after median and posterior tibial nerve stimulation using a bipolar surface electrode at the wrist or ankle, respectively. Two or three sets of 200–1000 responses were averaged, with either surface or subcutaneous electrodes for scalp derivations (Cc’-Fz’ for upper limbs and Cz’-Fz’/Cc’-Cz’/Ci’-Cc’ for lower limbs). Cortical responses (N20/P25 and P37/N45) were recorded in all cases. Normality was defined for N20 and P37 according to our laboratory database latencies, N20: 18.64 ms (SD 0.91) and P37: 38.12 ms (SD 2.44) [18]. Additionally, inter-side amplitude differences greater than 50% were evaluated.

#### 2.1.3. Transcranial Magnetic Stimulation

We performed transcranial magnetic stimulation (TMS) using a single pulse to stimulate the motor cortex (coil centered in Cz) and spinal root levels (cervical for UL and lumbar for LL), with peripheral recordings over the abductor pollicis brevis or abductor digiti minimi for UL and tibial anterior for LL. The central motor conduction time (CMCT) was calculated by subtracting the time needed for the signal to travel over the peripheral segment (spinal cord to the muscle) from the total latency (motor cortex to the muscle).

Normal CMCT, according to our laboratory database, was defined as <8.5 ms for UL and 16.5 ms for LL. Additionally, the levels of impairment were described as mild (CMCT 8.5–10 ms for UL, and 16.5–21.5 ms for LL), moderate (UL: 10–15 ms; LL: 21.6–25 ms), and severe (UL > 15 ms; LL > 25 ms), including a significantly decreased amplitude or absence of response.

### 2.2. Surgical Procedure

A laminectomy and durotomy adapted to the lesion level were performed in all the cases. Subsequently, a myelotomy was carried out following two approaches, either via DREZ using the methodology described by Sindou et al. [19], or the conventional midline approach between the posterior columns. Next, shunt placement or subarachnoid space reconstruction was performed for syrinx drainage. For the subarachnoid space reconstruction, a decompressive laminectomy–adhesiolysis with limited myelotomy, syringostomy, and introduction of a small synthetic dural graft GORE^®^ (W. L. Gore & Associates, Inc., Phoenix, AZ, USA) into the stoma that acted as a ‘stent’ to avoid myelotomy reclosure was carried out. The final step was an increase in spinal canal size with a wide duroplasty (GORE^®^).

### 2.3. Anesthesia

All the patients underwent general anesthesia. Upon arrival in the operating room, the patients were non-invasively monitored, including blood pressure, heart rate, electrocardiography, and pulse oximetry. For neuromonitoring of anesthetic depth, a Bispectral Index™ (BIS™) Monitoring System (Medtronic Covidien, Minneapolis, MN, USA) was placed on their forehead. Anesthetic induction was performed with fentanyl (2 mcg/kg), propofol (2 mg/kg), and a single dose of atracurium besylate (0.5 mg/kg) as a neuromuscular blocking agent only to allow orotracheal intubation. Following orotracheal intubation, end-tidal carbon dioxide (CO_2_), esophageal temperature, central venous pressure, diuresis, and continuous blood pressure through the radial artery were routinely monitored to maintain the mean blood pressure between 65 and 80 mmHg. Pulmonary volume-controlled ventilation was used with a fraction of inspired oxygen (FiO_2_) of 0.5, tidal volume of 6 to 7 mg/kg, respiration rate between 12 and 16 breaths per minute, and a positive end-expiratory pressure of 5 mmHg was used to achieve a partial arterial carbon dioxide pressure (PCO_2_) of approximately 35–45 mmHg. Anesthetic maintenance was performed under total intravenous anesthesia with propofol and remifentanil to maintain BIS values between 40 and 60, thus not interfering with the intraoperative neurophysiological monitoring.

### 2.4. Intraoperative Neurophysiological Monitoring

All the neurophysiological data were recorded with an Xltek^®^ Protektor32 (Natus Neurology, Inc., Middleton, WI, USA) following international guideline recommendations [5,20,21]. Open MEPs, SEPs, and free-running electromyography (EMG) baselines were recorded after anesthesia induction and before the skin incision in all cases. In addition, prepositional baselines were taken when needed, in which case, prepositional signals in the supine position were recorded and compared with post-positioned signals in the prone position. Mapping techniques were used selectively, depending on the surgical approach or the surgeon’s request.

#### 2.4.1. Somatosensory Evoked Potentials

Posterior tibial nerve (PTN) SEPs were elicited through electrical stimulation at the medial malleolus. The cortical potential was recorded via corkscrew electrodes (Natus Neurology, Inc.; Middleton, WI, USA) placed on the scalp using three different derivations: Cz’-Fz, Cz’-C3’, and C3’-C4’ after right stimulation and Cz’-Fz, Cz’-C4’, and C4’-C3’ after the left stimulation, according to the 10–20 international system. Ulnar or median nerve SEPs were elicited by stimulating the wrist and recording at the scalp using the following derivations: C3’-Fz for the right nerves and C4’-Fz for the left ones. For the stimulation, either surface or subcutaneous electrodes were used. Proximal and distal electrodes were assigned as the cathode and anode, respectively. A supramaximal stimulus was applied to activate myelinated sensory axons. All SEPs were collected bilaterally and continuously monitored throughout the surgery. The SEP alert criteria included >50% amplitude reduction of the cortical potential after ruling out technical and anesthetic considerations [20,22].

#### 2.4.2. Motor Evoked Potentials

Transcranial electrical stimulation of the motor area (via corkscrew electrodes placed at C1/C2 or C3/C4 according to the 10–20 international system) was performed by applying an anodal short train paradigm (5–7 pulses, 250 Hz, 500 µs per pulse and suprathreshold intensities). Recordings were performed via paired subdermal needle electrodes inserted in the muscles of the upper and lower extremities. At least the distal muscles of the upper and lower extremities were used bilaterally. According to the level of the surgery, different muscles were targeted, such as the trapezius, deltoids, biceps brachii, extensor digitorum, abductor pollicis brevis (APB), adductor digiti minimi (ADM), quadriceps femoris, tibialis anterior (TA), and abductor hallucis (AH). The MEPs were monitored intermittently after the main surgical maneuvers or at the surgeon’s request. The disappearance of response was considered a major criterion, and a >80% amplitude reduction was also reported as a minor criterion, according to McDonald et al. [23].

#### 2.4.3. D-Wave

In addition to MEPs, D-waves were used to monitor the motor pathway in some cases. D-waves were elicited by a single electrical pulse (0.5 ms duration) delivered transcranially at C1/C2 or C3/C4 and recorded via an epidural electrode placed caudally to the lesion. If possible, another D-wave recorded via another epidural electrode placed cranially to the lesion was used as a control. A 50% amplitude reduction criterion was applied [5,7].

#### 2.4.4. Free-Running Electromyography

Free-running EMG of the muscles of the upper and lower extremities described above was performed bilaterally. Trains of neurotonic EMG discharges were continuously observed and reported [24].

#### 2.4.5. Dorsal Column Mapping

The DC was identified using the phase-reversal technique described by M. Simon et al. [9,10]. After the SC was exposed, electrical stimulation (repetitive pulse at 3.7 Hz of frequency, 0.2 ms duration, up to 0.6 mA intensity) was applied to the SC using a hand bipolar probe. Cortical recordings were performed using corkscrew electrodes placed on the scalp at C3’-C4’ (10–20 international system). A positive cortical peak was recorded when the left DC was activated, and its specular image was observed when stimulating the right DC. However, no cortical response was obtained when the median raphe or DREZ were stimulated. Therefore, these areas could be marked as safe entry zones for the myelotomy. The DCM was performed throughout the entire area of the myelotomy before the incision.

#### 2.4.6. Root Mapping

After the nerve roots were exposed in the surgical field, they were electrically stimulated using a monopolar hand-held probe for identification and functional assessment of the root. The stimulated root elicited either a compound muscle action potential (CMAP) or an H response in the innervated muscles.

#### 2.4.7. Other Techniques

Additionally, control over the degree of peripheral relaxation was achieved through train-of-four (TOF) neurography with PTN stimulation at the internal retro malleolar level, and the CMAP was recorded in the abductor hallucis muscle. Finally, three bipolar EEG channels were recorded to control the depth of anesthesia.

## 3. Results

Ten surgical procedures were performed on nine patients with syringomyelia (two women and seven men, with a median age of 58 years, range: 31–76). The demographics, syringomyelia etiology and topography, preoperative examination, IONM events, and clinical outcomes for each patient are detailed in Table 1. Four patients presented with post-traumatic syringomyelia. One patient developed persistent syringomyelia secondary to Chiari malformation Type 1, despite posterior fossa reconstruction [25]. In one patient, the syringomyelia was related to spinal hemangioblastoma. In the remaining three patients, the syringomyelia etiology was a spinal arachnoid cyst, meningitis, and idiopathic. Cases 3 and 8 of Table 1 corresponded to two surgical procedures performed three years apart (2018 and 2021) on the same patient with post-traumatic syringomyelia due to a malfunction of the implanted shunt.

Mapping techniques were performed in all patients prior to the myelotomy incision for syrinx access. The dorsal nerve roots were stimulated and identified in the seven cases in which the myelotomy was performed through the DREZ. DCM was performed in four cases, with DCs successfully identified in three. Negative mapping was obtained when the median sulcus was stimulated in Cases 5 and 10 and the DREZ in Case 6. In Case 9, no responses were obtained, correlating with the presurgical damage to the DC at that level. After DCM, SEPs were continuously monitored during myelotomy in all cases, and no significant changes were observed.

A midline myelotomy was carried out in three cases, and a DREZ myelotomy was carried out in seven cases. A shunt for syrinx drainage was used in eight cases; five had a syringo-peritoneal shunt and the remaining three had a syringo-pleural shunt. Subarachnoid space reconstruction using a GORE^®^ patch was performed in two of the patients.

MEP baselines were obtained before surgical positioning in five cases (Cases 5, 6, 8, 9, and 10). In one case, bilateral loss of MEP responses—affecting muscles of the upper and lower extremities—was observed when the patient was placed in the prone position, which was recovered after adjustment of the neck position. No changes between pre- and post-positional recordings were observed in four cases. MEPs from distal muscles to the surgical entry point were obtained in all cases. In Case 4, MEPs from the lower extremities were bilaterally absent at the baseline. Therefore, distal upper extremity muscle MEPs were used as distal controls at the surgical level. Monitorable and bilateral SEP responses from the upper extremities were present in nine cases. SEPs were absent at baseline from the lower extremities in three cases bilaterally and three unilaterally, correlating with the findings of preoperative neurophysiological examinations (Table 2). D-wave recordings distal to the surgical level were recorded in two patients (Cases 5 and 7), with no significant changes being observed throughout the surgical procedure.

All monitored modalities remained stable throughout the surgery in six cases, correlating with no new postoperative neurological deficits. In two cases, significant attenuation or loss of MEPs was observed and recovered after a corrective surgical maneuver was applied. MEPs were preserved at the end of the surgery and no new postoperative deficits were observed. In two cases, a significant MEP decrement was noted, which did not recover by the end of the surgery. Both patients developed new postoperative motor deficits. No significant intraoperative SEP decrement was observed. In one patient (Case 5), a transitory EMG alert was reported, consisting of a long train of neurotonic discharges observed in the free-running EMG of the muscles corresponding to the segmental level of the surgery. The surgical plan was adjusted and there was no postoperative deficit.

**Figure 1:**
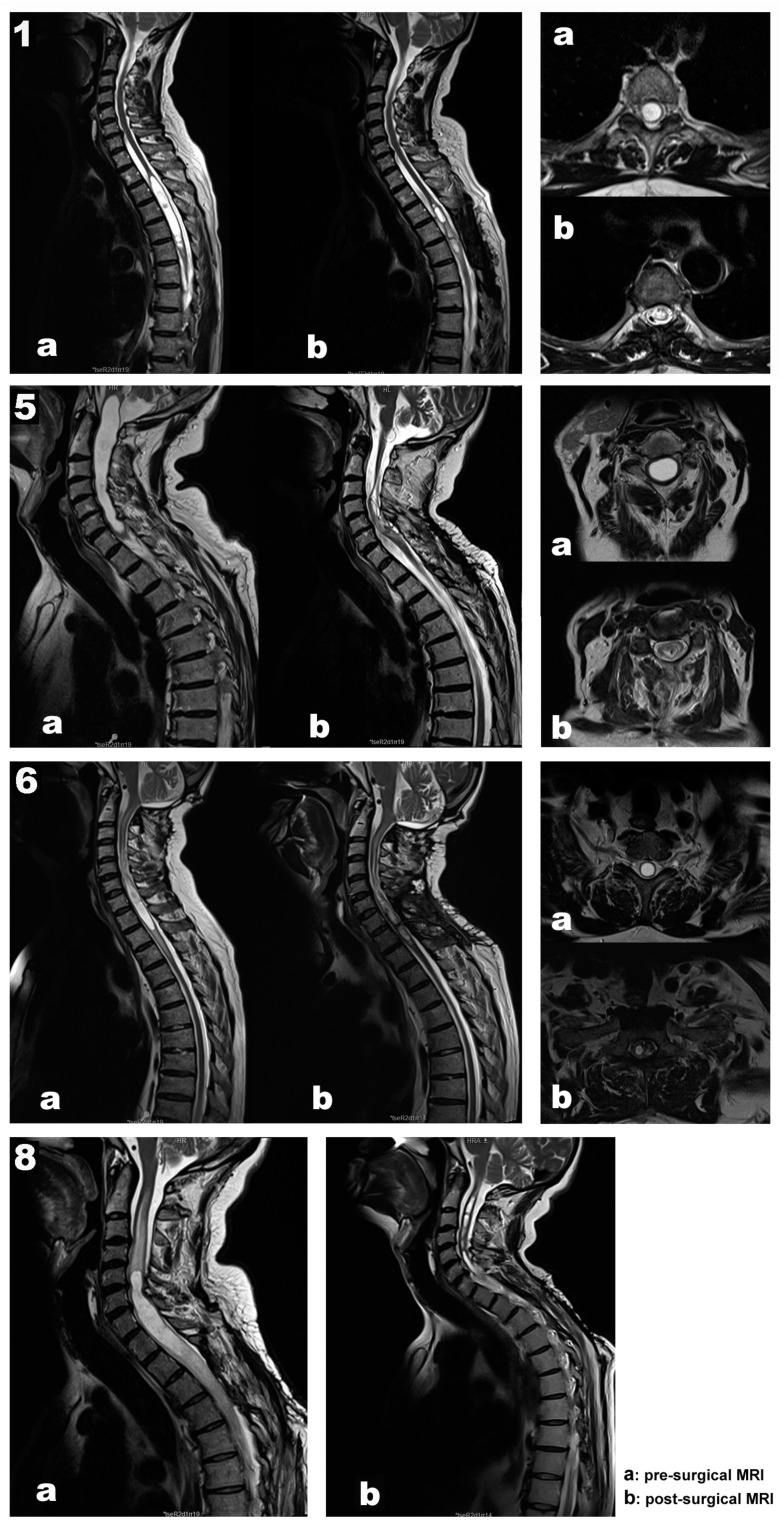
Preoperative (a) and follow-up (b) magnetic resonance image scans from each illustrative case (1, 5, 6, and 8). Sagittal and axial view of the syrinx.

Our data support a good correlation between the intraoperative signals at the end of the surgery and the postoperative neurological status. In addition, in all patients, the length and diameter of the syringomyelic cavities were reduced in the follow-up MRI performed six months after surgery (Figure 1, images b).

### Illustrative Cases

**Figure 2:**
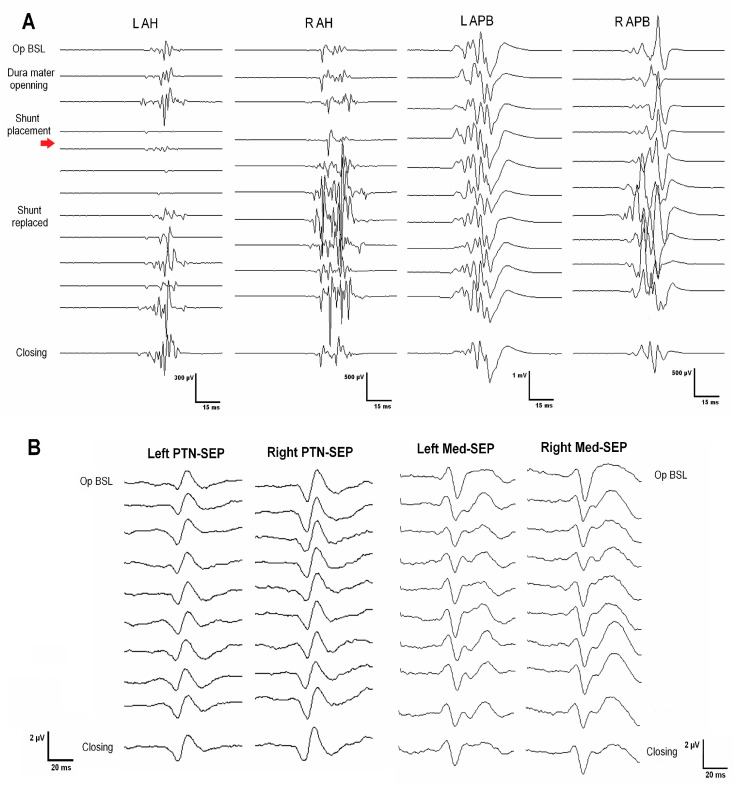
(**A**) Transitory significant decrement/loss of left adductor hallucis MEP after shunt placement (red arrow). This was recovered after slightly repositioning the catheter, while the contralateral MEP remained stable. (**B**) The somatosensory evoked potential from the posterior tibial and median nerve (as upper extremity control) remained stable throughout the procedure.

**Case 1.** A 67-year-old woman, independent for daily-life activities, presented with post-traumatic syringomyelia due to falling from a horse when she was 30 at the Th2–Th6 segments (Figure 1. Top panel, images a). She referred to back pain with approximately seven years of evolution and an associated loss of sensitivity and strength in the left lower extremity in the last year. At surgery, MEPs and SEPs from the four extremities were present at baseline. After the laminectomy, a left DREZ myelotomy was performed at the Th4–Th5 segment. A syringo-peritoneal shunt was placed. During the shunt placement, a significant reduction in amplitude of more than 80% and a near-complete loss of left distal MEP (AH) was observed and reported. After slightly repositioning the shunt, the left AH MEP amplitude recovered. No changes were observed in the contralateral muscles. Bilateral PTN SEPs remained stable (Figure 2). The patient showed signs of improvement postoperatively, and the neurophysiological test showed no damage to the CST (Table 2).

**Case 5.** A 70-year-old man presented with syringobulbia and central SC edema, which extended to Th1, associated with a spinal hemangioblastoma (Figure 1, second panel, image a). Clinically, the patient was presented with progressive proximal paresis in the four extremities, especially in the right upper extremity. In addition to the neurological impairment, neurophysiological tests showed acute radicular/anterior horn affectation. Moderate to severe axonal loss was associated with the right C8–Th1 segments, with minimal repercussions on the left side at the same level. Additionally, we found mild involvement of the DC pathway for the upper limbs and right lower limb and mild impairment of the pyramidal tract for the left lower limb, with normality for the right lower limb and both upper limbs.

**Figure 3:**
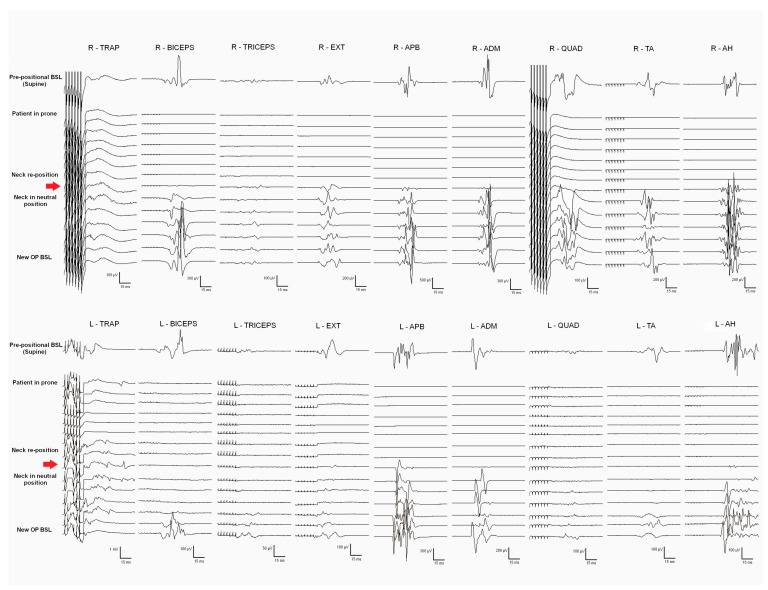
Muscle motor evoked potentials (MEPs) from the upper and lower extremities. Prepositional baselines were taken when the patient was supine (first trace). Monitorable responses were observed in all muscles (trapezius, biceps, extensor digitorum, abductor pollicis brevis, adductor digiti minimi, quadriceps femoris, tibialis anterior, and abductor hallucis, bilaterally). After the patient was turned into a prone position, MEP loss from all muscles (except the bilateral trapezius) was observed. After neck repositioning (the neck extension was decreased and the neck fixed in a neutral position), MEP responses recovered in all muscles (red arrow). After that, new opening baselines were taken before the skin incision.

General anesthesia was induced, and prepositional baselines were recorded while the patient was supine. Sensory and motor pathways of the SC, as well as segmental metameres, were intraoperatively monitored. Monitorable responses were observed in all the muscles recorded (trapezius, biceps brachii, extensor digitorum, APB, ADM, quadriceps femoris, TA, and AH, bilaterally). After the patient was turned to the prone position, an immediate loss of MEPs from all the muscles (except the bilateral trapezius) was observed. The surgical team modified the neck flexion, fixing the neck in a neutral position, and MEP responses fully recovered after neck repositioning (Figure 3). New baselines were taken before the skin incision.

**Figure 4:**
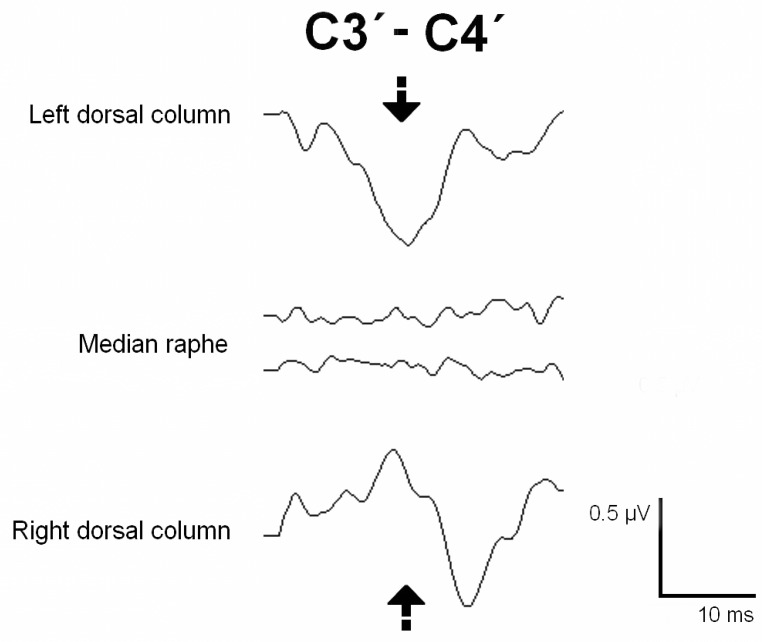
Dorsal column mapping using the phase-reversal technique. Before the myelotomy, the spinal cord surface was stimulated with a bipolar hand probe at 0.5 mA. The cortical somatosensory evoked potential (SEP) was recorded at the cortical C3’-C4’ derivation (10–20 International system). The Left and the right dorsal column were identified, as well as the medial raphe. A positive potential (top black arrow) was observed when the left dorsal column was stimulated, and a negative potential (bottom black arrow) was observed on the same cortical derivation when the right dorsal column was found. The points where no response was obtained were considered “negative mapping” and were marked as a secure entry zone. The entire myelotomy area was tested longitudinally.

A C5–Th1 laminectomy was performed, and after the dura mater was opened, an epidural catheter was placed caudally, obtaining a good-quality D-wave. DCM was performed to identify the DCs and the median raphe (Figure 4). The entire myelotomy area was tested longitudinally to find a safe entry zone. During the myelotomy, the PTN SEPs remained stable. The cavity was explored, and the possible hemangioblastoma was coagulated. A long train of EMG discharges was observed during catheter placement in the right extensor and triceps muscles. The catheter was removed, and a surgical break was given until the EMG discharges stopped. After the shunt replacement, another train of EMG discharges was observed in the same muscles. The shunt was finally removed, and the surgical plant was modified. The subarachnoid space was reconstructed with a GORE^®^ patch. The SEPs, MEPs, and D-waves were stable during the whole procedure. The patient woke up with no new neurological deficits and showed proximal strength improvement in the upper and lower extremities. The postoperative neurophysiological test showed CST conduction stability and improvement in the degree of denervation at the right segmental level of the surgery (Table 2).

**Case 6.** A 61-year-old man presented with a Chiari malformation type 1 that required posterior fossa reconstruction 13 years ago. Even though a new pseudo cisterna magna was created, the MRI showed persistent cervical–dorsal syringomyelia (Figure 1. Third panel, images a). Two years before surgery, the patient presented with clinical deterioration, spastic paraparesis, hyperreflexia in both lower extremities, a positive Babinski sign, and a dorsal column syndrome with a positive Romberg. In the last few months, the patient used a wheelchair to move around, both inside and outside the home, having difficulties maintaining a standing balance. In the left upper limb, there was global paresis, with a muscular strength of 3 out of 5 that was more accentuated in the extensor muscles of the hand and fingers, with marked amyotrophy of the thenar and hypothenar eminences. MEPs and SEPs from the four extremities were present at the baseline. The patient’s positioning was monitored, establishing prepositional MEP and SEP baselines while the patient was supine. No significant changes were observed in the prone position. A laminectomy at the C7–Th1 level was performed. After the dura mater was opened, the left DC was identified using the DCM technique, and no response was obtained when the DREZ was stimulated. In addition, the left C8 dorsal root was stimulated and identified with a monopolar hand probe. A 4 mm lateral myelotomy was performed in segments C7–Th1 at the level of the left DREZ, and a syringo-pleural shunt was placed. The SEPs and MEPs were stable throughout the procedure, and no EMG discharges were observed. In the postoperative period, the patient’s paresis of the left upper extremity improved, presenting better muscle balance in the extensors of the hand and fingers.

**Case 8.** A 48-year-old male presented with recurrent post-traumatic holo-cord syringomyelia (Figure 1. Bottom panel, image a). The patient had a traffic accident in 2012, presenting incomplete acute SC injury with sensory level L3 right/Th11 left, motor level L4 right/L2 left, and ASIA D. Initially, the patient underwent posterior fusion at Th11–L3 segments in 2012. However, he developed secondary syringomyelia that required several surgical treatments, with significant clinical deterioration since September 2014. Previous surgeries included a Th6–Th8 DREZ myelotomy for intramedullary stent placement in 2016, a DREZ myelotomy at C2–Th4 for a syringo-pleural shunt in 2017, and a left DREZ myelotomy at C7–Th1 for syringo-peritoneal derivation in 2018 (Case 3 in Table 1). Over the last years, the patient reported neuropathic back pain, thermo-algesic sensory alteration in the left hemithorax and hemiabdomen, and progressive deterioration of strength and sensitivity in the extremities. Before surgery, the patient experienced progressive clinical worsening, with bilateral upper extremity weakness that was more prominent in the distal muscles. The MRI showed progression of the syringomyelic cavity, with greater extension at the cranial level, reaching the medullary obex. The patient also presented marked post-laminectomy cervical-thoracic kyphosis, so it was decided to correct the deformity and replace the syringomyelic shunt in the same surgery (Case 8 in Table 1).

No changes in the baseline recordings were observed when the patient was placed in the prone position. At skin opening, monitorable SEPs were recorded from both upper extremities and the right lower limb and were absent from the left lower extremity. MEPs were recorded in all monitored muscles except the left AH. The MEPs and SEPs remained stable during instrumentation for the posterior arthrodesis at the C5–Th11 level. A laminectomy was performed at the C5–Th1 level. The previous syringo-peritoneal tube was located and sectioned without observing CSF flow. Dural opening was conducted with wide resection of the fibrotic layer above the previous pleural and peritoneal shunts. After identifying the left C7 and C8 nerve roots, a 5 mm myelotomy was performed in the left DREZ until the septated syrinx was entered. The dentate ligaments were sectioned bilaterally, observing CSF leakage through the dural ridges. After hemostasis, the dura mater was left open. Immediately after placement of the new catheter, an abrupt fall in left MEPs was observed, involving the ADM, APB, rectus femoris, and TA. Unfortunately, these potentials did not recover at the end of the surgery. The patient presented with immediate postoperative global weakness in the left extremities― predominantly affecting the lower extremity―and numbness of the left upper limb. The sensory and motor symptoms of the left upper extremity progressively improved in the following days, with the persistence of weakness in the left lower limb.

## 4. Discussion

Syringomyelia is a complex and diverse entity, with particularities in its pathophysiology and surgical approaches. Therefore, a specific IONM protocol is essential to prevent and minimize possible spinal cord damage associated with the surgical intervention. IONM plays a critical role in guiding the surgeon to locate a safe entry zone for myelotomy, minimizing DC damage, and detecting and avoiding sensory and corticospinal pathway injuries by adapting the surgical approach.

The concept of syringomyelia drainage was first described in 1892 by Abbe and Coley; however, it remains a controversial issue in neurosurgery today due to the high complication rates reported (approximately 50% in some series) and the frequent need for additional reoperations (20%) after shunt placement [26]. Furthermore, any intervention over the syringomyelic cavity, such as shunt placement or syringostomy, requires a myelotomy and poses a significant risk of additional iatrogenic SC injury. In addition, the SC of these patients is usually already compromised before surgery, and the anatomical references are severely disturbed by the presence of the syrinx, which displaces and distorts neural structures.

Despite the high risk of iatrogenic injury in syringomyelia surgery, there is little evidence to recommend a specific IONM protocol for this type of surgery. To our knowledge, only one article has been published about the benefits of IONM in this specific surgical field, and only MEPs and SEPs were included. In line with the findings of Pencovich et al. [27], our data presented a good correlation between the neurophysiological tests conducted preoperatively and the baselines established at the time of surgery, as well as good monitorability data for MEPs and SEPs. In the series presented by this group, the only patient who had a transient intraoperative decrease in MEPs had mild postoperative worsening of symptoms, even though the signals recovered to basal levels before the end of the surgery. In our study, the two transient MEP events were observed, which recovered after a corrective maneuver and did not correlate with any new postoperative deficit, neither clinically nor in postoperative neurophysiological exams. Despite the limitation of the number of cases, it represents an excellent correlation with the postoperative clinical status.

We present ten cases of syringomyelia surgery with IONM associated with different underlying conditions. Despite the severe SC involvement that all patients presented with preoperatively, we observed a high level of monitorability. MEPs from distal muscles to the surgical level were obtained in all cases. Monitorable SEP responses from the upper extremities were present in nine cases and from the lower extremities in seven cases. Most patients had damaged long fibers of the sensory system, as shown in pathological baseline responses with long latencies and low amplitudes. Therefore, it requires additional efforts to achieve monitorable baseline recordings. However, distal responses at the surgical level were obtained in 80% of cases. The three patients for whom no monitorable SEP responses were obtained from the lower limbs (Cases 4, 8, and 9) already presented an absence of cortical potential in the preoperative study (Table 2). These findings demonstrate an excellent correlation between the preoperative neurophysiological examination and the intraoperative baseline recording at the start of the surgery.

The three transient events observed intraoperatively were relevant (Case 5: global MEP deterioration related to neck flexion in the prone position and EMG neurotonic discharges associated with shunt placement; Case 1: transitory loss of left AHB MEPs during shunt placement through the left DREZ). In all of these cases, intraoperative signals recovered after a corrective maneuver, such as neck repositioning, catheter removal, or redirection of its trajectory, respectively. At the end of the surgery, no significant changes were observed compared to the baseline recordings, and the patients did not present any new postoperative deficits. Additionally, the postoperative neurophysiologic test showed CST conduction stability in both patients and even an improvement in the degree of denervation at the segmental level of surgery in Case 5 (Table 2). These observations show the potential reversibility of impending SC damage when an iatrogenic insult is detected in real time, before permanent injury is established. Similarly, the two patients with persistent intraoperative MEP loss presented with a postoperative clinical decline that consistently correlated with the decline demonstrated in the postoperative tests (Table 2).

Prepositional baselines are critical to detect potential damage to corticospinal and posterior DC tracts in the prone position. Ensuring a safe neck position is essential when the cervical levels and the medulla are involved. The benefits of IONM in correct patient positioning have been previously reported in cervical and Chiari malformation spinal surgeries [28,29,30,31]. In our series, Case 5 presented with global MEP deterioration in the prone position, which fully recovered when the neck was adjusted to a more neutral position (Figure 3). The consequences of not immediately detecting such extensive damage to the corticospinal pathway would have been devastating.

IONM of the functional integrity of SC pathways is crucial to detect and prevent surgical injury, which monitoring techniques like MEPs and SEPs can achieve. However, some surgical steps require identifying the functional SC tracts, which is the role of the mapping methods [31]. It has been proven that the accuracy of IONM increases when multiple modalities are simultaneously monitored [13,32,33]. In our series, DCM using the gracilis SEP phase-reversal technique was used to identify a safe zone entry for the myelotomy, protecting the functional DCs in four cases. In Case 5, both DCs were identified, and negative mapping was obtained at the medium raphe over the entire myelotomy area. In Case 6, the left DC was stimulated and identified, and negative mapping was observed in the DREZ. Only left DC stimulation evoked a response in Case 10, whereas the right DC was silent, indicating dislocation or non-functioning. In Case 9, no responses were obtained despite ruling out technical issues. Negative mapping is also valuable, as it can guide the myelotomy toward the non-functioning area.

Our study supports multimodal IONM as the standard of care in syringomyelia surgery. In our opinion, the IONM protocol should include MEP to monitor the corticospinal pathway at the segmental level and long tracts, SEP to monitor the somatosensory pathway, and free-running EMG for the identification of neurotonic discharges associated with mechanical irritation of CST fibers, since that continuous monitoring techniques have been crucial in our series to detect imminent injuries and avoid permanent spinal cord injury through corrective maneuvers. D-wave monitoring, if possible, would be advocated as complementary to MEPs for long-term prognosis values in the case of MEP loss. Mapping techniques are highly recommended and should be adapted to the surgical procedure and the patient´s condition. For example, in our series, DCM was essential for identifying the posterior cords and a safe entry zone for the myelotomy in the four cases where the technique was used. This technique could be applied in the two surgical approaches described for the myelotomy before the incision: in the posterior median sulcus and through the DREZ. Similarly, CST mapping may be useful before and during myelotomy via DREZ due to the anatomical proximity to the CST. However, due to a technical limitation, our series could not analyze its benefit. These mapping techniques afford the surgeons relevant information to identify and potentially preserve functional fibers. Root mapping could be added for identification and functional assessment of the root. However, its protective role does not seem very relevant. Additionally, monitoring the patient’s positioning using prepositional baseline recordings can be critical when syringes involve the cervical SC and medulla.

## 5. Limitations

Our study is a retrospective review with a small number of cases. Additionally, CST mapping was not performed due to a technical limitation of our monitoring equipment, which did not allow for stimulation with the double-train paradigm. In addition, not all the techniques could be applied to all the patients because the personal expertise or technical equipment necessary to perform the DCM was not available in the initial cases (cases 1–4). Finally, the postoperative neurophysiological tests could not be performed immediately after surgery in all cases, and the period between surgery and testing varied between cases (especially delayed in Cases 6 and 7, where neurophysiological examinations were performed two years after surgery).

## 6. Conclusions

Multimodal intraoperative neurophysiological monitoring, including monitoring and mapping techniques, is feasible, correlates well with the postoperative clinical outcome, and may help prevent iatrogenic SC damage during syringomyelia surgery. In our series, the DCM was useful to guide a safe entry zone for myelotomy, and the continuous IONM techniques were crucial to detect impending injuries and avoid permanent damage by adapting surgical maneuvers.

## Figures and Tables

**Table 1:** Clinical and surgical features of the patients.

Case No.	Age	Gender	Type	Syrinx Location	Surgery	Pre-Operative Clinical Examination	IOMN Modalities	IOMN Finding and Events	Clinical Outcome
1	67	Female	Post-traumatic	Th2–Th6	Th4–Th5 laminectomy Left DREZ myelotomy Syringoperitoneal shunt	Loss of sensation of the left LL associated with left LL weakness	MEPs SEPs Free-EMG Root mapping	Transitory loss of left AHB MEP recovered after shunt repositioning SEPs remained stable throughout surgery Root identification	No new deficits Subjective clinical improvement
2	44	Male	Post-traumatic	Holocord	C7–Th2 laminectomy Right DREZ myelotomy Syringopleural shunt	Gait disturbances Muscle weakness	MEPs SEPs Free-EMG Root mapping	Root identification Significant decrement of LLs MEP (R > L) after shunt placement, not recovered SSPEs remained stable throughout surgery	Worsening Temporary paraplegia Worsening of distal strength in right LL
3	48	Male	Post-traumatic	Holocord	Reintervention (2 previous surgery without IONM) C7–Th1 laminectomy Left DREZ myelotomy Syringoperitoneal shunt	Sensory disturbances on the left hemithorax, hemiabdomen, and left UL LLs pain	MEPs SEPs Free-EMG Root mapping	Root identification SEPs and MEPs remained stable throughout surgery	Pain improvement Transitory left UL improvement lasting some weeks Some months later, strength worsening
4	31	Male	Post-traumatic	C5–Th1	C5–C7 laminectomy Left DREZ myelotomy Subarachnoid space reconstruction	Right UL weakness Subjective sensory disturbances on ULs	MEPs SEPs Free-EMG Root mapping	LL SEPs absent baselines Root identification SEPs and MEPs remained stable throughout surgery	No new deficits Improvement of the sensory disturbances on ULs Stable right UL weakness
5	70	Male	Hemangioblastoma related	Medulla–C7	C5–Th1 laminectomy Midline myelotomy Subarachnoid space reconstruction	Paresthesias in ULs Ataxia Mild muscle weakness in LLs	MEPs SEPs Free-EMG D-wave DCM	Transitory MEP decrement after prone position, recovered after neck reposition DCM for median raphe identification Transitory EMG discharges upon shunt placement Stable MEPs, SEPs, and D-wave	No new deficits Proximal UL and LL strength improvement, which persists one year later
6	61	Male	Chiari malformation	C6–Th2	C7-Th1 laminectomy Left DREZ myelotomy Syringoperitoneal shunt	Progressive paraparesis Gait disturbances	MEPs SEPs Free-EMG DCM Root mapping	Prepositional baselines remains stable DCM for left DC and safe zone entry identification at DREZ Root identification SEPs and MEPs stable throughout surgery	No new deficits Distal left UL strength improvement One year later, progressive strength and sensory worsening
7	74	Male	Idiopathic	C2–Th8	Th1–Th3 laminectomy Left DREZ myelotomy Syringopleural shunt	Ataxia, progressive left LL spasticity, and LL weakness Right UL pain	MEPs SEPs Free EMG D-wave Root mapping	Root mapping MEP, SEP, and D-wave stable throughout surgery	No new deficits LL strength improvement Reduction of spasticity and pain
8	48	Male	Post-traumatic	Holocord	Reintervention (3 previous surgeries) C5–Th1 laminectomy Left DREZ myelotomy Syringoperitoneal shunt	Muscle weakness No more data is available	MEPs SEPs Free-EMG Root mapping	Prepositional baselines stable Root C7 and C8 mapping Sudden loss of left MEPs (ADM, APB, TA, and recto femoris) after shunt placement, not recovered by the end of the surgery	Worsening Hemiparesis (LLs > ULs)
9	76	Female	Spinal arachnoid cyst	Th7–conus	Th8–Th9 laminectomy Midline myelotomy Syringoperitoneal shunt	LLs weakness Ataxia	MEPs SEPs Free-EMG DCM	Prepositional baselines stable DCM (no responses) LL SEP absent baselines MEPs stable throughout surgery	Slight worsening of the ataxia Initially, neuropathic pain worsens; after a few months, similar neuropathic pain to the pre-operative condition
10	57	Male	Post-meningitis	Medulla–holocord	Th6–Th8 laminectomy Midline myelotomy Syringopleural shunt	Cervico-dorsal myelitis Slight weakness in the left UL LLs paraparesis	MEPs SEPs Free-EMG DCM	LL SEP absent baselines DCM (identification of left DC and medial sulcus. Not response at right DC) SEPs and MEPs stable throughout surgery	No new deficits Left UL strength improvement

DC: dorsal column; DCM: dorsal column mapping; EMG: electromyography; IONM: intraoperative neuromonitoring; LL: lower limb; MEP: motor evoked potential; SEP: somatosensory evoked potential; UL: upper limb. Cases 3 and 8 correspond to two surgical procedures performed on the same patient three years apart (2018 and 2021) due to a malfunction of the implanted shunt.

**Table 2:** Pre- and post-operative neurophysiologic test results of the patients.

Case		Preoperative	Postoperative	Electrodiagnostic Outcome
1	EMG	Upper limbs not explored. Normal LL	Upper limbs not explored	Unknown
	SEP	Mild impairment of dorsal columns in both LL (normal UL)	Mild impairment of dorsal columns in both LL (UL not explored)	Stable
	TMS	Normal pyramidal tract conduction in both LL (UL not explored)	Uninjured pyramidal tract in both LL (UL not explored)	Stable
2	EMG	Right UL: complete denervation at C8/D1 level and severe at C5–C7 Left UL: almost complete denervation C5–C8/D1 (brachial plexopathy)	Not explored	Unknown
	SEP	Severe impairment of dorsal columns in left UL (absent cortical potential) and moderate in both LL (normal right UL)	Moderate impairment of dorsal columns in both LL (UL not explored)	Stable for LL. Unknown for UL
	TMS	Normal pyramidal tract conduction in both LL (UL not explored)	Mild impairment of pyramidal tract in both LL (UL not explored)	Deterioration of LL CST. Unknown for UL
3	EMG	Mild acute denervation at C5–C7 left levels. Severe acute denervation at C8 and L3–S1 left levels. Mild chronic denervation at C7–C8 and L5 right side levels.	Not explored	Deterioration of left roots/anterior horn
	SEP	Moderate impairment of dorsal columns in left LL and mild in right LL. Normal for both ULs	Stable for right LL, moderate impairment for left LL (amplitude decrement of cortical potential). Mild impairment for both ULs at the cervico-medullary level, with normal cortical conduction.	Stable, mild changes for left LL and cervico-medullary level.
	TMS	Uninjured pyramidal tract in right UL and LL. Severe impairment on the left side (absence of responses for left LL)	Stable for right limbs. Persistent abolition of motor cortical response for UL and LL from the left side	Stable
4	EMG	Bilateral moderate–severe chronic denervation at C7–C8/D1 levels	Bilateral moderate–severe chronic denervation at C7–C8/D1 levels	Stable
	SEP	Severe impairment of dorsal columns for both LL (absent cortical potential). UL not explored	Mild impairment of dorsal columns for right UL. Left UL and both LL not explored	Unknown
	TMS	Severe impairment of pyramidal tract for both LLs. ULs not explored	Not explored	Unknown
5	EMG	Moderate–severe acute denervation at right C8/D1 level and mild left C8/D1	Mild chronic denervation at bilateral C8/D1 level	Improved degree of denervation at right C8/D1. Stable for the left side
	SEP	Mild impairment of dorsal columns in both upper limbs and right lower limb (normal left LL)	Mild impairment of dorsal columns in both upper limbs and right lower limb (normal left LL)	Stable
	TMS	Mild impairment of pyramidal tract in left LL (normal right LL and both UL)	Mild impairment of pyramidal tract in left LL (normal right LL and both UL)	Stable
6	EMG	Severe chronic denervation at left C5–C8 levels and mild–moderate at right C6–C7	Severe chronic denervation at left C7 level and moderate left C5–C6 (right UL not explored)	Improved degree of denervation at left C5–C6. Unknown evolution for right UL.
	SEP	Mild impairment of dorsal columns in right UL and left LL; moderate impairment in left UL and right LL	Severe impairment in left UL, moderate in right LL, and mild in right UL and left LL	Moderate deterioration of left UL dorsal column
	TMS	Mild impairment of pyramidal tract in right UL and LL, moderate in left UL (normal left LL)	Mild impairment of pyramidal tract in right UL and LL and moderate in left UL and LL	Global mild deterioration for CST
7	EMG	Not explored	Not explored	Unknown
	SEP	Moderate impairment of dorsal columns for both LL (left > right) at the cervical–lumbar segment. Mild impairment for both ULs	Moderate impairment of dorsal columns for LLs at the cervical–lumbar segment. Mild–moderate impairment for both ULs	Global mild deterioration of dorsal columns conduction
	TMS	Severe impairment of pyramidal tract for left LL (absence of potential). Mild–moderate impairment for right LL. Normal for both ULs	Not explored	Unknown
8	EMG	Not explored	Not explored	Unknown
	SEP	Moderate impairment of dorsal columns in left LL, mild in right LL. Mild impairment for both ULs at the cervico-medullary level	Severe impairment of dorsal columns in left LL (absence of cortical potential) Mild in right LL. Mild–moderate impairment for both ULs at the cervico-medullary level (right > left)	Deterioration of left dorsal column conduction
	TMS	Moderate impairment of pyramidal tract in left UL. Severe impairment for left LL (absence of potential). Normal for right UL and LL	Severe impairment (absence of potential) of the pyramidal tract in left UL. Mild impairment for right UL and left LL. Uninjured pyramidal tract in right LL	Deterioration for right UL CST. Improvement for left LL CST.
9	EMG	Not explored	Not explored	Unknown
	SEP	Severe impairment of dorsal columns for both LL (absent cortical potential). ULs not explored	Severe impairment of dorsal columns for both LL (absent cortical potential). Normal function for both ULs	Stable
	TMS	Mild impairment of pyramidal tract for both LLs (left > right) ULs not explored	Not explored	Unknown
10	EMG	Moderate–severe acute denervation at left C5–C8/D1 levels Mild–moderate on the right side. Bilateral moderate–severe acute denervation at L3-S1 levels	Not explored	Unknown
	SEP	Mild impairment of dorsal columns for left UL, normal for right UL. Severe impairment of dorsal columns for both LL (absent cortical potential)	Not explored	Unknown
	TMS	Mild impairment of pyramidal tract for left UL, moderate for right UL and severe for both LLs	Not explored	Unknown

CST: Corticospinal tract; LL: lower limb; UL: upper limb. Normal pyramidal tract conduction: CMCT ≤ 16.5 ms for tibialis anterior, CMTC ≤ 8.5 ms for abductor pollicis brevis/ADM.

## Data Availability

Not applicable.
